# *Streptococcus*, the Predominant Bacterium to Predict the Severity of Liver Injury in Alcoholic Liver Disease

**DOI:** 10.3389/fcimb.2021.649060

**Published:** 2021-03-17

**Authors:** Xiaodan Zhong, Ping Cui, Junjun Jiang, Chuanyi Ning, Bingyu Liang, Jie Zhou, Li Tian, Yu Zhang, Ting Lei, Taiping Zuo, Li Ye, Jiegang Huang, Hui Chen

**Affiliations:** ^1^ Geriatrics Digestion Department of Internal Medicine, The First Affiliated Hospital of Guangxi Medical University, Nanning, China; ^2^ Guangxi Key Laboratory of AIDS Prevention and Treatment, School of Public Health, Guangxi Medical University, Nanning, China; ^3^ Life Science Institute, Guangxi Medical University, Nanning, China; ^4^ Nursing College, Guangxi Medical University, Nanning, China

**Keywords:** alcoholic liver disease, gut microbiota, hepatic function, *Streptococcus*, aspartate aminotransferase

## Abstract

**Background:**

New evidence implies that the imbalance of gut microbiota is associated with the progression of alcoholic liver disease (ALD) and that the composition of gut microbiota is altered in ALD patients. However, the predominant bacterium in patients involved in the progress of ALD has not been identified. The purpose of this study is to investigate the predominant bacterium in the early and end-stages of ALD as well as the relationship between the bacterium and the degree of liver injury.

**Methods:**

We enrolled 21 alcoholic fatty liver (AFL) patients, 17 alcoholic liver cirrhosis (ALC) patients and 27 healthy controls, and sequenced the 16S rRNA gene of their fecal microbiota. The gut microbiota composition and its relationship with the indicators of clinical hepatic function were assessed using canonical correspondence analysis (CCA), spearman correlation heatmap and multivariate association with linear (MaAsLin) Models.

**Results:**

The composition and structure of gut microbiota changed greatly in different stages of ALD, and the degree of disorder was aggravated with the progression of ALD, even in the early stage. Moreover, the relative abundance of *Streptococcus* was highly enriched only in patients with ALC (P <0.001), and positively correlated with AST level (P = 0.029). The abundance of *Streptococcus* distinguished the liver injury of ALC patients from the controls with an area under the receiver-operating characteristic curve (AUC) of 0.877 (P < 0.001).

**Conclusions:**

These findings indicate that the imbalance of gut microbiota exists at the early and end-stages of ALD, and the degree of disorder is aggravated with the progression of ALD. *Streptococcus*, as the predominant bacterium, may be a microbiological marker to evaluate the severity of liver injury in ALD patients.

## Introduction

Alcoholic liver disease (ALD) is a chronic liver disease caused by long-term alcohol-use disorder, and its pathological changes include steatosis, steatohepatitis, fibrosis, cirrhosis, and hepatocellular carcinoma (Dunn and Shah, 2016).Worldwide, alcohol-related liver diseases are the leading cause of liver disease-related mortality in most countries (Savolainen et al., 1993; Gao and Bataller, 2011). Although it is known that the amount and mode of alcohol consumption are certain risk factors for the onset of ALD (Stickel and Hampe, 2012), the pathogenesis of ALD is not fully understood. Recent studies have provided evidence that ALD may be related to oxidative stress injury from alcohol metabolism, abnormal methionine metabolism, injury of inflammatory mediators, intestinal microbiota imbalance and bacterial translocation (Shao et al., 2018).

The gut microbiota, also named intestinal microbiome or intestinal microbiota, plays an important role in the metabolism and immune regulation of the host (Burkholder and McVeigh, 1942; Chung et al., 2012; McDermott and Huffnagle, 2014; Spasova and Surh, 2014; Morrison and Preston, 2016). The gut microbiota contributes to host physiology through the production of a myriad of metabolites (Krautkramer et al., 2021). As the main product of gut microbiota, short-chain fatty acids (SCFA) have been found to play an important role in lipid turnover and energy homeostasis by reducing adipocyte lipolysis and adipogenesis (Hong et al., 2005). Furthermore, SCFA, in particular propionate and butyrate, have also been shown a strong anti-inflammatory effect through downregulating of lipopolysaccharide (LPS)-induced cytokines expression (Nastasi et al., 2015). Once the gut microbiota is out of balance, the important pathogenic substances produced by the maleficent bacteria enter the liver *via* the portal vein and then cause liver damage (Szabo, 2015).

Previous studies have shown that alcohol abuse leads to alterations in the structure and composition of gut microbiota and promotes the occurrence and progression of ALD (Sarin et al., 2019). The imbalance of gut microbiota happens both in alcohol-related human disorders and mice models (Yan et al., 2011; Leclercq et al., 2014). Intestinal metabolism of alcohol produces a high concentration of toxic acetaldehyde that alters the intestinal barrier and promotes LPS translocation (Tang et al., 2008; Malaguarnera et al., 2014). Acetaldehyde and LPS then induce Kupffer cells to release ROS, proinflammatory cytokines and chemokines that contribute to hepatocyte damage (Ceni et al., 2014).

It is also found that the degree of dysbiosis in gut microbiota is closely related to the severity of ALD. The liver function test is often used to assess liver function in the clinic, and ALD patients are usually identified by increasing levels of liver enzymes such as aspartate aminotransferase (AST) and gamma-glutamyltranspeptidase (GGT). In particular, the indexes are elevated with the severity of ALD. It’s also reported that the abundance of Akkermansia muciniphila was reduced in patients with alcoholic steatohepatitis compared with healthy controls, which was indirectly correlated with the severity of hepatic disease (Grander et al., 2018). However, the correlation between these predominant bacteria in gut microbiota and abnormal indicators of hepatic function in ALD patients is still unclear, and its significance remains to be elucidated.

In this study, we investigated the change of structure and composition of gut microbiota in patients at the early and end-stages of ALD. Furthermore, the correlation between the indexes of the clinical liver function examination and the bacterial community was analyzed. The aim of this study was also to identify the predominant gut bacterium of ALD patients, which could be related to the progression of the disease and used as a microbiological marker to assess the severity of ALD.

## Materials and Methods

### Ethics Statement

This study was approved by the Ethics Committee of Guangxi Medical University, Nanning, Guangxi, China. All the study subjects provided written informed consent, and volunteered to participate in the investigation for scientific research.

### Patient Recruitment

Due to the higher susceptibility to ALDs in women than in men (Dunn and Shah, 2016), only males (who are more tolerant to alcohol) were chosen as participants in this study. The ALD patients in this study were separated into two groups: alcoholic fatty liver (AFL) and alcoholic liver cirrhosis (ALC), and enrolled from Department of Gastroenterology of the First Affiliated Hospital of Guangxi Medical University if they satisfied the following criteria: 1) long-term alcohol consumption history or drink more than 40 g per day, 2) clinical imageological examination (ultrasonic B or CT) showed steatosis or cirrhosis, 3) no antibiotics or probiotics were used at least two weeks before fecal sample collection. In addition, the patients who met the following criteria were excluded from the study: 1) if the patients were suffering from viral hepatitis, drug hepatitis, autoimmune liver disease or other specific etiology of liver disease, 2) if the patients were suffering from the diseases that confirmed related to microbiome, such as inflammatory bowel disease, gastrointestinal tumors, heart disease, diabetes and so on, 3) if the patients regularly use or recently use specific drugs, which may affect the condition of gut microbiota, such as proton pump inhibitor (PPI), laxatives and so on.

Healthy controls were selected from the Physical Examination Centre of the First Affiliated Hospital of Guangxi Medical University and met the following standards: 1) did not have a long-term alcohol consumption history or drank less than 40 g per day, 2) no abnormalities in the clinical imageological examination (ultrasonic B or CT), 3) no antibiotics or probiotics were used at least two weeks before fecal sample collection, 4) matched with the patients by age and gender. The exclusion criteria mentioned above is also applied to the healthy controls.

### Fecal Sample and Basic Data Collection

Fresh stools were collected from the IC patients in the morning after their admission to the hospital, and from the healthy subjects during their physical examination. The fecal samples should be put in stool collection tubes with stool DNA stabilizer (Stratec, Berlin, GER), and stored in -80°C lab freezers immediately. All the study subjects’ general information, including past medical history, medication history, past surgical history, bowel habit and so on, was acquired from our questionnaire. And the results of clinical image examination and the indicators of hepatic function were collected from the participants’ medical report.

### DNA Extractions and Amplification of 16S rRNA

Genomic DNA was extracted from fecal samples using the EZNA^®^ DNA Kit (Omega Bio-tek, Norcross, GA, USA) by a tissue lyser (Bao et al., 2017), following the standard protocol. PCR amplification was performed with the 338F (5’-ACTCCTACGGGAGGCAGCAG-3’) and 806R (5’-GGACTACHVGGGTWTCTAAT-3’) primers, which targeted the V3–V4 region of the bacterial 16S rRNA gene. Amplified products were detected by 1% agarose gel electrophoresis and purified using the AxyPrep DNA Gel Extraction Kit (Axygen Biosciences, Union City, CA, USA).

### 16S rRNA Sequencing and Microbiota Analysis

Sequencing was conducted by Shanghai Majorbio Bio-pharm Technology (Shanghai, China). The purified amplicons were pooled and paired-end sequenced (2 × 300) on an Illumina MiSeq platform (San Diego, CA, USA) according to the manufacturer’s instructions. The sequencing was removed using the following criteria: 1) sequences shorter than 200 bp, 2) ambiguous bases, 3) sequences with an average mass less than 25. Sequences were assigned to operational taxonomic units (OTUs) with 97% similarity. The sequences were classified using the SILVA database containing bacterial and fungal ribosomal RNA sequences. The raw reads were deposited into the NCBI Sequence Read Archive (SRA) database (**BioProject ID: PRJNA690835**).

The community diversity of gut microbiota was analyzed after the sequence data subsampled by minimum sequence numbers. In addition, partial least squares discriminant analysis (PLS-DA) and analysis of similarities (ANOSIM) were also performed to compare the bacterial composition among groups. Linear discriminant analysis (LDA), coupled with effect size measurement (LEfSe) analysis, was conducted to elucidate the differences of bacterial taxa between groups (Segata et al., 2011). The relationships between gut microbial community structure and the indicators of hepatic function were analyzed by canonical correspondence analysis (CCA), spearman correlation heatmap and multivariate association with linear (MaAsLin) Models. CCA eliminates redundant variables depending on other measured variables, automatically selecting variables with large effects, and on the variance inflation factor values to gradually remove redundant parameters, and the significance levels are based on 999 Monte Carlo permutations.

### Statistical Analysis

All results were presented as mean ± standard deviation (SD) or median (interquartile range), depending on whether they fit the normal distribution. The one-way ANOVA test, nonparametric tests and receiver operating characteristic (ROC) curve were performed using SPSS version 26.0. The Wilcoxon rank-sum test was used for alpha diversity analysis, including Shannon, Chao and Ace indexes. And Kruskal–Wallis H test was used to compare the relative abundance of gut microbiota among groups. Results with P <0.05 between groups were declared statistically significant.

## Results

### Grouping Situation and Data Output

A total of 27 healthy controls, 21 alcoholic fatty liver (AFL group) and 17 alcoholic liver cirrhosis (ALC group) participants were enrolled for stool sample and clinical data collection (**Table 1**). To profile the differences in the composition of gut microbiota at different stages of ALD, the V3 region of bacterial 16S rRNA gene from 65 samples were sequenced. After quality trimming and chimaera checking, we obtained 3,308,525 valid sequences in total. Based on the 97% similarity, unique representative sequences were classified into 1486 operational taxonomic units (OTUs) through a clustering operation, from which, 30 phyla, 625 genus and 1065 species were detected.

**Table 1:** Patient characteristics.

Patient characteristics	Healthy Control (n = 27)	Alcoholic fatty liver (AFL, n = 21)	Alcoholic liver cirrhosis (ALC, n = 17)
**Age**	48.7 ± 10.8	45.4 ± 8.3	55.9 ± 9.6
**Sex (male/female)**	27/0	21/0	17/0
**Body mass index (BMI)**	25.0 ± 2.6	26.2 ± 2.5	23.2 ± 3.6
**TBiL**	11.0 ± 4.0	11.0 ± 4.0	20.9 (9.8,42.5)
**DBiL**	2.0 ± 0.7	2.6 ± 1.4	8.2 (3.9,25.3)
**IBiL**	9.0 ± 3.9	10.0 ± 5.2	11.9 (5.2,22.6)
**DB/TB**	0.2 ± 0.1	0.2 ± 0.1	0.5 ± 0.1^a,b^
**TP**	75.1 ± 2.9	74.7 ± 3.7	65.5 ± 8.7^a,b^
**ALB**	46.4 ± 2.0	46.7 ± 2.6	30.1 ± 5.0^a,b^
**GLO**	28.7 ± 4.1	28.0 ± 3.8	32.8 ± 10.9
**A/G**	1.7 ± 0.3	1.7 ± 0.3	0.9 ± 0.3^a,b^
**GGT**	25 (19,33)	48 (33,77)	173 (49,439)^a^
**AST**	20.1 ± 3.9	26.0 ± 7.6^a^	54 (41,79)^a,b^
**ALT**	20.5 ± 6.4	31 (19,41)	23 (17,41)
**AST/ALT**	1.1 ± 0.3	0.9 ± 0.6	2.1 (1.3,3.2) ^a,b^

Data represent as mean ± standard deviation (SD) or median (interquartile range). Statistical analyses were performed with one-way ANOVA test or nonparametric tests. a, compared to control group, *P < 0.05. b, compared to AFL group, *P < 0.05.

Analysis of similarities (ANOSIM) was performed based on Bray -Curtis distances (***P < 0.001). It showed that the difference between the groups was greater than that within groups, which indicated that the grouping method, based on the processes of ALD, was effective and meaningful.

### The Community Diversity of Gut Microbiota in ALD Patients Declined

**Figure 1:**
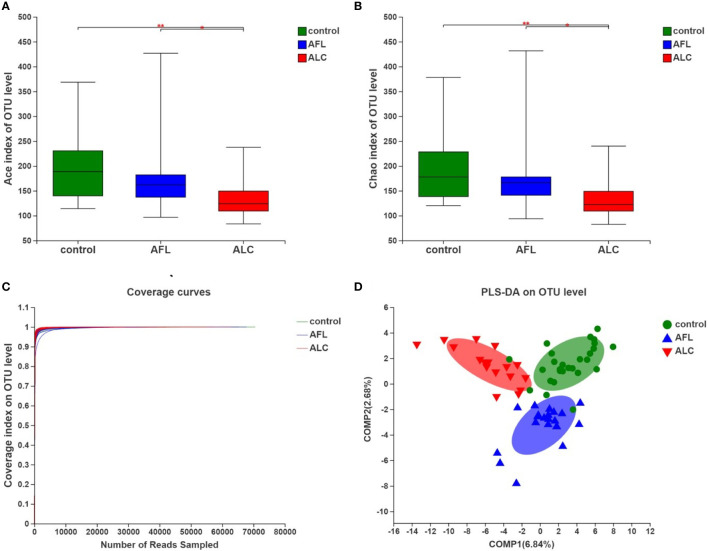
The community diversity of gut microbiota in patients with alcoholic fatty liver (AFL) or alcoholic liver cirrhosis (ALC) compared with healthy controls (control). **(A)** The Ace diversity indexes, **(B)** The Chao diversity indexes, **(C)** Coverage curves on OTU level, and **(D)** PLS-DA on OTU level. *P < 0.05, **P < 0.01.

Alpha diversity refers to the diversity of a specific area or ecosystem, of which, Chao and Ace are commonly used to estimate the total number of species of gut microbiota. As shown in **Figures 1A, B** and **Table 2**, there were significant differences in the estimators of OTU richness indices including Ace (139.86 ± 46.57 vs. 192.96 ± 62.27, P = 0.004) and Chao (140.69 ± 46.01 vs. 192.22 ± 62.92, P = 0.005) between ALC and control groups. Furthermore, there were also significant differences in Ace and Chao indexes between ALC and AFL groups. The Chao index in ALD patients decreased gradually, indicating that the imbalance of gut microbiota was aggravated with the development of the disease. In addition, the coverage curves of each group eventually flattened out (**Figure 1C**), showing that the sequencing data of each group approached saturation point and the data at the OTU level could cover most bacteria in the intestinal tract.

**Table 2:** Sequencing data summary and community diversity.

	Control	AFL	ALC
**OTUs**	172 ± 55	174 ± 85	123 ± 40^a,b^
**Shannon**	2.99 ± 0.51	2.88 ± 0.48	2.87 ± 0.50
**Ace**	192.96 ± 62.27	190.24 ± 88.56	139.86 ± 46.57^a,b^
**Chao**	192.22 ± 62.92	189.82 ± 87.60	140.69 ± 46.01^a,b^

Data represent as mean ± standard deviation (SD) Statistical analyses were performed with Wilcoxon rank-sum test. a, compared to control group, *P < 0.05. b, compared to AFL group, *P < 0.05..

To evaluate the similarity of the gut microbiota communities among different samples within the groups, partial least squares discriminant analysis (PLS-DA) was performed at the OTU level. The samples of each group were clustered, and the grouped ellipses of three groups on PLS-DA were separated (**Figure 1D**). It suggested that there were significant differences in the overall structures of the gut microbiota communities in different periods of ALD.

### The Difference Analysis of Composition in Gut Microbiota

**Figure 2:**
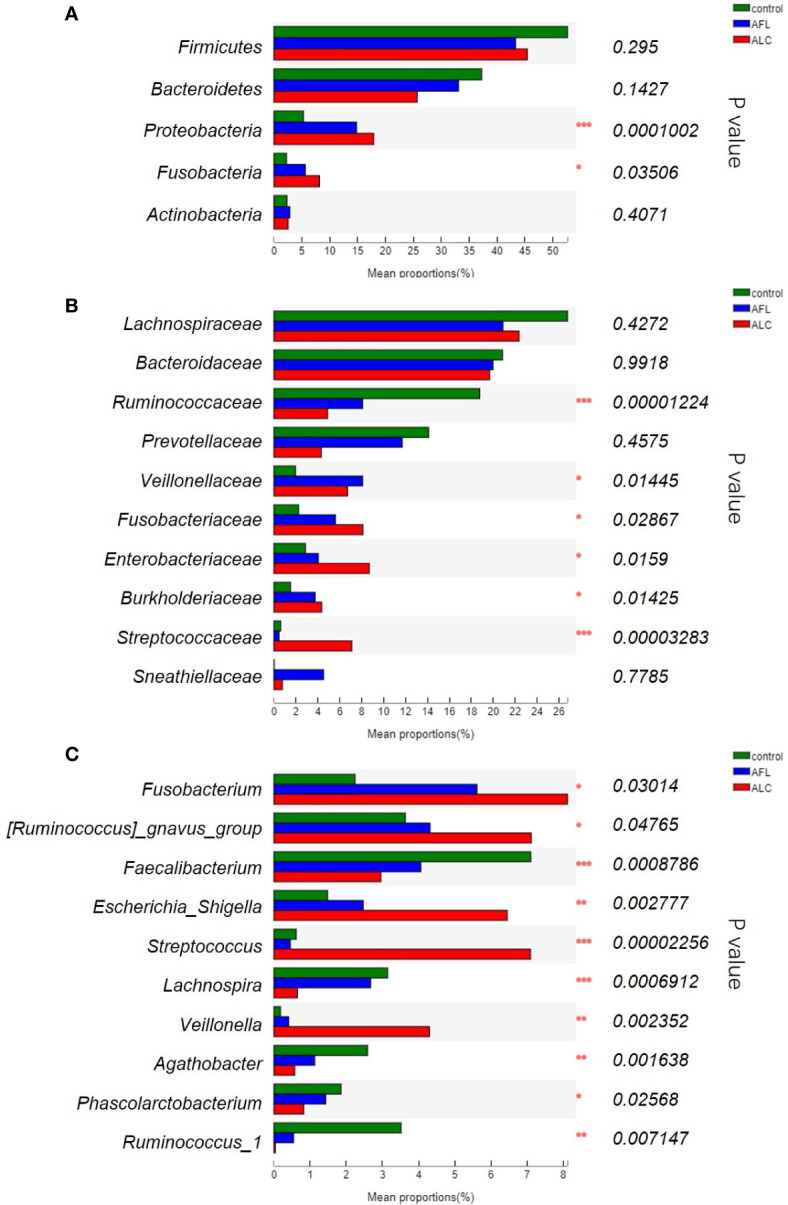
The relative abundance of the gut microbiota between three groups. **(A)** At the phylum level, **(B)** at the family level, **(C)** significant differences between bacteria at the genus level. Statistical analysis was performed by the Kruskal–Wallis H test. *P < 0.05, **P < 0.01, ***P < 0.001.

Except for the differences of the structures in gut microbiota, the composition of bacterial communities in patients with fatty liver or cirrhosis was indeed different from healthy controls. The most abundant bacterial phylum in the different groups was shown in **Figure 2A**. We found that *Proteobacteria* gradually enriched in patients as the disease progressed (***P <0.001), as well as *Fusobacteria* (*P <0.05). However, the relative abundance of *Bacteroidetes* showed a protracted downward trend among the three groups.

We further compared the relative abundance of gut microbiota of the three groups at family levels. As shown in **Figure 2B**, the relative abundance of *Ruminococcaceae* decreased in ALD patients was lower than that in the healthy controls (***P <0.001), while *Fusobacteriaceae*, *Enterobacteriaceae* and *Burkholderiaceae* (*P <0.05) was increased in the AFL and ALC groups. *Streptococcaceae* (***P <0.001) were remarkably enriched in the ALC group only.

At the genus level, the top ten predominant bacteria with statistical differences in three groups were shown in **Figure 2C**. The relative abundance of *Faecalibacterium* (***P <0.001), *Lachnospira* (***P <0.001), *Agathobacter* (**P <0.01) and *Ruminococcus_1* (**P <0.01) in the AFL and ALC groups decreased as the disease progressed, while *Fusobacterium* (*P <0.05) and *Escherichia-Shigella* (**P <0.01) increased gradually. Similarly, *Streptococcus* (***P <0.001) and *Veillonella* (**P <0.01) were significantly enriched in the ALC group only.

**Figure 3:**
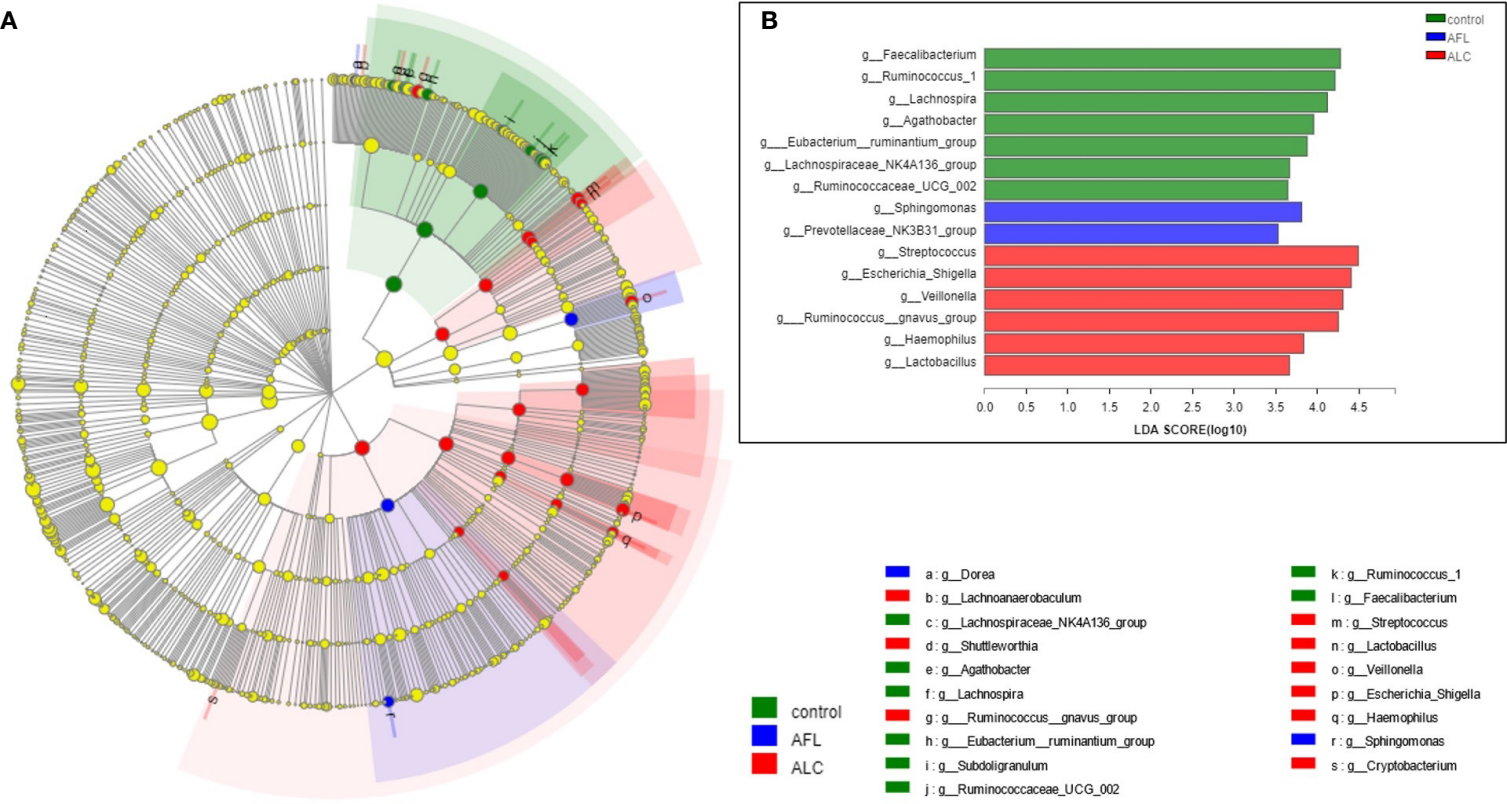
Microbial taxa identified in different groups using LEfSe analysis. **(A)** Cladogram showed the phylogenetic distribution of bacteria that were significantly enriched between the three groups. Different colors spots represent significantly enriched bacterial in the corresponding group. **(B)** LDA scores showed significant differences in bacterial within groups at the genus level.

Through analyzing the structure and composition of gut microbiota among the three groups, we implemented the linear discriminant analysis (LDA) effect size (LEfSe) method to further validate the specialized communities within the groups. As shown in **Figure 3A**, a cladogram displayed all the significantly enriched bacterial structure at phylum and genus levels. LDA scores of 3.5 or above were confirmed by LEfSe (**Figure 3B**), indicating that the listed bacteria were the dominant bacterial community in each group. At the genus level, *Faecalibacterium*, *Sphingomonas* and *Streptococcus* were the most significant enrichment bacteria in the healthy control, AFL and ALC group, respectively.

### The Correlation Between Relative Abundance of Bacterial and Clinical Hepatic Function Indexes

From clinical indexes of hepatic function in each group (**Table 1**), we found that ALC patients were associated with abnormal clinical indicators of hepatic function, such as direct bilirubin/total bilirubin (DB/TB), total protein (TP), albumin (ALB), the ratio of albumin to globulin (A/G), AST, GGT and aspartate aminotransferase/alanine aminotransferase (AST/ALT). The hepatic function abnormalities were characterized by damaged hepatic cells and impaired liver function. Meanwhile, AST, ALT and GGT were also elevated in AFL patients, indicating that ALD patients at early stage might have hepatocyte damage without any clinical symptoms.

Usually, the liver function impairment in ALD patients was assessed through clinical hepatic function upon biochemical examination. AST and GGT were the main abnormal indicators of clinical hepatic function in ALD patients. Therefore, we studied whether the indicators of the clinical laboratory tests were related to microbial community structure. After using variance inflation factor (VIF) analysis to filter suitable clinical indexes, we selected CCA, based on a single peak model to verify the relationship between samples, clinical indexes and gut microbiota.

**Figure 4:**
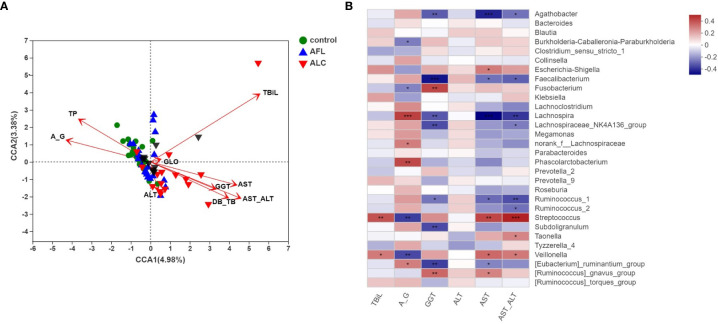
Correlation analysis of the structures of gut microbiota and clinical factors. **(A)** CCA analysis on genus level showed the correlations between the bacterial community structures and the indicators of clinical laboratory tests. **(B)** Heatmap analysis of the correlation between the top 30 predominant bacteria at genus level and the clinical factors. Red spots represent a positive correlation, while blue spots represent a negative correlation. *P < 0.05, **P < 0.01, ***P < 0.001.

As shown in **Figure 4A**, the red arrow represents the indexes of the clinical liver function test, and its length means the extent to which this index affects the gut microbiota. Total bilirubin (TBiL), A/G, TP, AST/ALT, DB/TB, GGT and AST were significantly associated with the bacterial community structure. As the indicators reflected the severity of impaired liver function, AST/ALT, DB/TB, GGT, AST were significantly related to ALC patients (red inverted triangles), and the species of intestinal bacteria in genus level, marked in black triangles, were closely linked with the clinical indexes, including *Bacteroides*, *Ruminococcus*, *Faecalibacterium* and *Streptococcus*.

Then, we utilized a heat map to show the association between microbial species at the genus level and the selected clinical factors (**Figure 4B**). It’s worth noting that *Streptococcus*, as a significantly enriched bacteria in the ALC group, was positively correlated with TBiL, AST and AST/ALT, while negatively correlated with A/G. The results reflected that the relative abundance of *Streptococcus* is closely associated with the indicators of hepatic function.

### *Streptococcus* Correlated with Liver Clinical Factor, which might be an Indicator to Evaluate the Severity of Liver Injury

**Figure 5:**
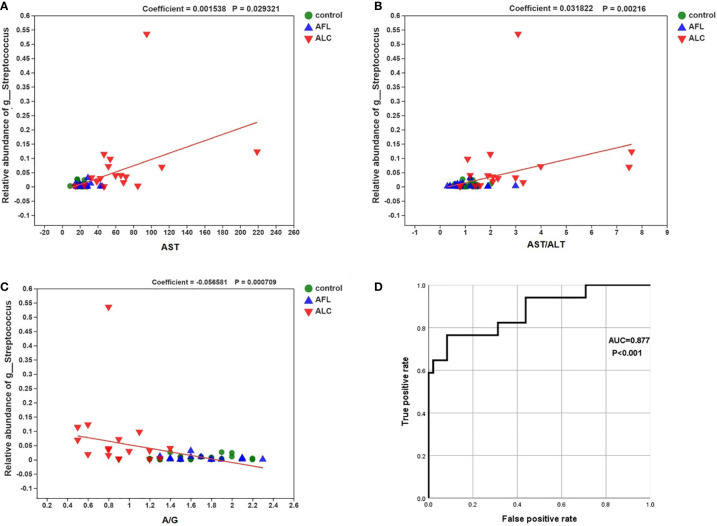
Correlations between the relative abundance of *Streptococcus* and the level of the specific clinical factor. The correlations were calculated in MaAsLin. **(A)** AST (coefficient = 0.0015, p = 0.029), **(B)** AST/ALT (coefficient = 0.0318, p = 0.002) and **(C)** A/G (–0.0566, p = 0.0007). **(D)** the ROC curve about the relative abundance of *Streptococcus* and ALC.

MaAsLin analysis was used to analyze the relationship between the relative abundance of *Streptococcus* and individual specific clinical factor, such as AST, AST/ALT and A/G. The relative abundance of *Streptococcus* was positively correlated with the levels of AST and AST/ALT, while negatively correlated with A/G in the ALD patients (**Figures 5A–C**; coefficient = 0.0015, p = 0.029; coefficient = 0.0318, p = 0.002; coefficient = –0.0566, p = 0.0007, respectively).

Finally, we performed a receiver operating characteristic curve (ROC) and calculated the area under the curve (AUC) to evaluate the diagnostic efficiency of the relative abundance of *Streptococcus* on the liver function of ALC patients. The results showed that AUC was close to 1 (**Figure 5D**; AUC = 0.877, P < 0.001), suggesting that it is probable to assess the severity of liver injury in ALC patients by the relative abundance of *Streptococcus*.

## Discussion

Recently, numerous studies have suggested that the progression of alcoholic liver disease is associated with dysbacteriosis (Sarin et al., 2019), although the relationship between the altered gut microbiota and the severity or progression of ALD is still unknown. We compared the structure and composition of gut microbiota in the early and end-stages of ALD patients and found that the community diversity and composition of gut microbiota in ALD patients were changed with disease progressed. Moreover, we discovered the linear correlation between the predominant bacterium and the main indicators of hepatic function in ALC patients. Through the correlation analysis, we inferred a new potential key species, which was integrally involved in the progression of ALD.

Generally speaking, ALD patients have elevated serum aminotransferase levels. However, in the early stage of ALD, patients’ serum aminotransferase levels can be normal. ALT, AST and TBil are sensitive indexes to evaluate the degree of liver injury among many liver function indexes. It is well known that chronic alcohol consumption can lead to elevated serum GGT (Conigrave et al., 2003; Hietala et al., 2005). GGT is a sensitive marker of alcohol consumption and liver dysfunction, which could return to normal after 2–3 weeks of abstaining from drinking (Niemela, 2016). We found that AST, ALT and GGT were slightly elevated in AFL patients, while almost all the indicators were abnormal in ALC patients, except GGT, suggesting that AFL patients possibly have hepatocyte damage caused by alcohol consumption, and that ALC patients have sustained impaired liver function even after abstinence from alcohol.

Growing evidence suggests that one of the manifestations of dysbiosis in gut microbiota is the changed community diversity. Similar to previous studies (Addolorato et al., 2020; Zheng et al., 2020), we also found that the alpha diversity and beta diversity of the gut microbiota in ALD patients were changed, even in its early stages, which showed that the richness of species decreased and the structure was altered due to alcohol consumption. Previous studies have reported that alcohol-induced changes in gut microbiota composition and metabolic function may contribute to alcohol-induced oxidative stress and the subsequent development of ALD (Ceni et al., 2014), meaning that alcohol abuse may be a main factor leading to the imbalance of gut microbiota.

Moreover, as the severity of ALD changes with time, so does the composition of gut microbiota and the relative abundance of bacteria. We found that *Enterobacteriaceae* and *Streptococcaceae* families were significantly increased in fecal samples from ALC patients, while *Ruminococcaceae* families were significantly reduced. Bajaj et al. pointed out that the change of the abundance of “good” vs. “bad” bacteria in liver cirrhosis patients was reflected by the cirrhosis dysbiosis ratio, which is determined by the abundance of *Lachnospiraceae* and *Ruminococcaceae* divided by the abundance of *Enterobacteriaceae* and *Bacteroidaceae* (Bajaj et al., 2014). As mentioned above, the changed abundance of the bacterium at the family level in three groups reduced the cirrhosis dysbiosis ratio, meaning the lower the ratio, the more serious the imbalance of gut microbiota in ALC patients. Furthermore, the specific bacterial families (*Ruminococcaceae*, *Streptococcaceae*, *Fusobacteraceae*) are strongly associated with cognition and inflammation in liver cirrhosis with hepatic encephalopathy (Bajaj et al., 2012).

Unlike some frequently reported bacterium, *Streptococcus* was observed to be the predominant gut bacterium of ALD patients. Previous studies have shown that *Streptococcus* was characteristically higher in liver cirrhosis and alcoholics (Chen et al., 2011; Tsuruya et al., 2016). And coincidentally, duodenal dysbiosis showed a dominance shift toward specific potential pathogenic bacteria genera (*Streptococcus*) in alcohol use disorders (Maccioni et al., 2020). In our study, we demonstrated that the relative abundance of *Streptococcus* was positively correlated with AST, which, as a major abnormal indicator of alcoholic liver injury, is typically more than twice that of ALT (Diehl, 2002). It revealed that the increased abundance of *Streptococcus* was correlated with the severity of hepatocyte damage in ALC. Similarly, Streptococcus bovis bacteremia has been associated with gastrointestinal diseases, especially colon cancer (Alvarez et al., 2015). According to multifaceted evidence that *Streptococcus* was closely related to liver cirrhosis, we propose a bold hypothesis that the abundance of *Streptococcus* could be used as an indicator to forecast the severity of cirrhosis. The ROC curve and AUC effectively proved the feasibility of this hypothesis. The greater the AUC means the stronger accuracy of the new bioindicator. Coincidentally, a core gut microbiota signature can identify cirrhosis across geographically separated cohorts, independent of other influencing factors on the gut microbiota (Oh et al., 2020). Therefore, a specificity of bacteria in gut microbiota, as a non-invasive diagnostic test for cirrhosis, has a sound theoretical basis, and the early diagnosis through the detection of changed bacterium is necessary, because of difficult diagnosis at the early stage of ALD.

Altogether, we demonstrated that the degree of imbalance of gut microbiota is more severe as the progression of ALD worsens, and the relative abundance of *Streptococcus* is related to the important indicators of clinical hepatic function. We can look forward that *Streptococcus* may become a microbial marker to evaluate the severity of liver injury in liver cirrhosis patients. As a potential pathogenic bacteria genera, *Streptococcus* was positively related to tryptophan, whose metabolites have been proved to be related to fat metabolism and proinflammatory reaction (Krishnan et al., 2018; Oh et al., 2020). Therefore, we hypothesis that the enriched *Streptococcus* possibly could cause hepatocyte damage by the proinflammatory effect of its metabolites.

## Data Availability Statement

The data sets presented in this study can be found in online repositories. The names of the repository/repositories and accession number(s) can be found below: BioSample database, BioProject ID: PRJNA690835 (https://www.ncbi.nlm.nih.gov/sra/PRJNA690835).

## Ethics Statement

Written informed consent was obtained from the individual(s) for the publication of any potentially identifiable images or data included in this article.

## Author Contributions

HC, JH, and LY designed the study. XZ and PC contributed to data analysis and wrote the paper. JZ, LT, YZ, TL, and TZ participated in data acquisition. JJ, CN, and BL participated in interpreting the results. All authors contributed to the article and approved the submitted version.

## Funding

This study was supported by the National Natural Science Foundation of China (NSFC, 81460305, 82002134, 81660334, 82060366), Youth Science Foundation of Guangxi Medical University (GXMUYSF201826), Guangxi Natural Science Foundation (2018GXNSFAA050099, 2017GXNSFAA198190), Guangxi Bagui Scholar (to JJ), Guangxi Medical University Training Program for Distinguished Young Scholars (to JJ).

## Conflict of Interest

The authors declare that the research was conducted in the absence of any commercial or financial relationships that could be construed as a potential conflict of interest.
